# Impact of Genotyping of *Mycobacterium tuberculosis* on Public Health Practice in Massachusetts

**DOI:** 10.3201/eid0811.020316

**Published:** 2002-11

**Authors:** Ann C. Miller, Sharon Sharnprapai, Robert Suruki, Edward Corkren, Edward A. Nardell, Jeffrey R. Driscoll, Michael McGarry, Harry Taber, Sue Etkind

**Affiliations:** * Massachusetts Department of Public Health, Jamaica Plain, Massachusetts, USA; † Harvard University, Boston, Massachusetts, USA; ‡ New York State Department of Health, Albany, New York, USA

**Keywords:** Public health, tuberculosis, epidemiology, polymorphism, restriction fragment length, DNA fingerprinting, epidemiology, molecular

## Abstract

Massachusetts was one of seven sentinel surveillance sites in the National Tuberculosis Genotyping and Surveillance Network. From 1996 through 2000, isolates from new patients with tuberculosis (TB) underwent genotyping. We describe the impact that genotyping had on public health practice in Massachusetts and some limitations of the technique. Through genotyping, we explored the dynamics of TB outbreaks, investigated laboratory cross-contamination, and identified Mycobacterium tuberculosis strains, transmission sites, and accurate epidemiologic links. Genotyping should be used with epidemiologic follow-up to identify how resources can best be allocated to investigate genotypic findings.

Genotyping of Mycobacterium tuberculosis isolates is accepted as a useful tool in many public health settings (1–13). Conventional epidemiology may be supported by analysis of isolates that cluster genetically by using IS6110 fingerprinting methods. Genotyping can confirm or disprove previously known epidemiologic connections or suggest unsuspected associations. We explore the impact that genotyping has had on public health practice in Massachusetts by investigating the following premises. Genotyping plays an indirect role in interrupting the transmission of tuberculosis (TB) by identifying unexpected epidemiologic links and unsuspected sites of transmission. By identifying and confirming outbreaks and their impact and aiding the investigation of laboratory cross-contamination, genotyping determines whether epidemiologic links found through conventional contact investigations represent true cases of transmission. This determination helps coordinators of TB-control programs to direct and evaluate their program activities.

Before Massachusetts become one of seven sentinel surveillance sites in the National Tuberculosis Genotyping and Surveillance Network (genotyping network), M. tuberculosis isolates were sent by the Massachusetts Department of Public Health, Division of Tuberculosis Prevention and Control (TB Division) to the regional laboratory (New York State Department of Health’s Wadsworth Center in Albany, New York) for genotyping on an as-needed basis. During the genotyping network study (July 1, 1996–December 31, 2000), one isolate from each new patient whose culture was positive for M. tuberculosis was sent for genotyping (14).

## Methods

### Laboratory Methods

Cowan and Crawford (15) and Crawford et al. (16) describe the laboratory protocol and design and methods of the genotypying network. The Wadsworth Center performed IS6110 restriction fragment length polymorphism (RFLP) and spoligotyping according to standardized procedures (16–18).

### Genotype Cluster Identification

The Centers for Disease Control and Prevention (CDC) developed a standard protocol for cluster investigations. For IS6110 strains with more than six copies in their fingerprint patterns (high-copy strains), a single cluster designation was allocated if two or more patients had isolates with identical RFLP patterns. Strains with six or fewer copies in their patterns (low-copy strains) were assigned a single cluster designation if two or more patients had both identical RFLP and spoligotype patterns. The cluster investigation by the genotyping network took place from January 1, 1998, to December 31, 2000.

### Epidemiologic Investigation of Clusters

To identify sources of transmission, we defined an epidemiologic link as two persons who shared space or time. For example, if a cluster included three persons (the source [A] and two subsequent patients [B and C]), the epidemiologic relationships identified for inclusion in the database would be A to B and A to C. However, when a common source was determined from outside the study, each relationship was counted only once without reference to direction (B and C, representing one link with no reference to A). An “expected link” was one found through conventional contact investigation, and an “unexpected link” was identified as a result of an RFLP cluster investigation.

To determine epidemiologic links, we reviewed medical records from health departments, hospitals, and clinics for each genotypically clustered case. Patients or their proxies were interviewed. Collected data included medical information (i.e., tuberculin skin-test results and BCG vaccine status, potential exposures, and previous diagnoses), demographics, and a 2-year social and work history.

CDC collaborated with the genotyping network sentinel sites in developing forms for data collection, patient consent, and interviews. The Massachusetts Department of Public Health institutional review board approved the protocol and forms. The patient consent form was translated into Spanish, Haitian Creole, Portuguese, Khmer, Vietnamese, and Chinese. Interpreters were present during interviews, when needed.

### Laboratory Cross-Contamination

For the period 1994–1997, laboratory cross-contamination or other error was suspected if more than one specimen processed at the same time was positive for M. tuberculosis. At the time, we considered that contamination was less likely to occur between specimens processed in different batches on the same day; therefore, this type of contamination was not addressed in the study. Contamination in different batches was, however, addressed in a broader 1998 genotyping network study on laboratory cross-contamination. Data and methods from that study are described elsewhere (19).

## Results

### Transmission Dynamics and Public Health Implications

From July 1, 1996, to December 31, 2000, a total of 1,281 TB cases were counted in Massachusetts. Of these, 1,043 (81%) were culture confirmed as TB. Positive cultures were obtained from 1,032 patients in Massachusetts, and 95% (984) of the isolates obtained from these patients were genotyped. The remaining isolates were not genotyped for various reasons, including inability to obtain growth when subcultured, contamination with other bacteria or fungi, and inability to acquire isolates from outside laboratories. Of the 984 isolates, 776 (79%) had high-copy strains (seven or more IS6110 copies in their patterns); 712 (72%) had unique DNA fingerprints; 272 (28%) aggregated into 82 clusters. Of 208 cases with low-copy strains, 100 (48%) met the study definition of genotype cluster.

### Links Established by Conventional Contact Investigation

Overall, 129 expected epidemiologic relationships in Massachusetts were identified. Of these, 37 relationships were between a person in the study and a person without a genotyped isolate (e.g., children, clinical patients). Of 92 relationships analyzed (88 persons), 67 (72%) demonstrated exact or similar (±1 hybridizing band) RFLP matches. Twenty-five relationships between 38 persons were not supported by genotyping, i.e., the isolates had different genotypes.

The 25 expected relationships not supported by genotyping were as follows: 11 in persons staying at the same homeless shelter at the same time; 5 in household contacts; 2 in nonhousehold family members, 3 in friends and social contacts, and 4 in co-workers. The five relationships involving household contacts were in persons from countries with a high prevalence of TB. These relationships included roommates from Mali, siblings from Kenya and Ethiopia, and parents and adult children from China and India.

### Additional Epidemiologic Links in RFLP Cluster Investigation

In addition to the 129 epidemiologic relationships identified before RFLP results, 11 unexpected epidemiologic relationships involving 21 persons were identified. We also identified additional and some previously unrecognized places of transmission (Table). Several of these are believed to have been caused by either casual contact or unsuspected settings. For example, cluster 5 in the table consisted of three patients who ostensibly had nothing in common. However, RFLP cluster investigation established that one patient had been the hairdresser of the second patient, who was a college student. The third patient was a security guard who had worked in a college dormitory frequented by the second case. At the onset of the guard’s illness, he was working at another facility.

**Table:** Settings of transmission for unexpected epidemiologic links within genotyped clusters

Cluster designation (no. IS*6110* copies*)*	No. unexpected epidemiologic relationships (persons)	No. expected relationships (persons)	Settings of transmission for unexpected epidemiologic links
1 (10)	1 (2)	2 (3)	Prison
2 (7)	1 (2)	2 (3)	Neighborhood, same public housing
3 (15)	1 (2)	0 (0)	Long-term care facility
4 (8)	1 (2)	0 (0)	Fast food restaurant
5 (12)	2 (3)	0 (0)	Hair salon, college building
6 (1)	1 (2)	0 (0)	Buddhist temple
7 (5)	1 (2)	0 (0)	Community barbecue^a^
8 (8)	1 (2)	1 (2)	Bars
9 (12)	1 (2)	0 (0)	Neighborhood, same markets
10 (9)	1 (2)	0 (0)	Neighborhood

^a^Community barbecue was held at different sites.

### Extent of an Outbreak in a Homeless Shelter

Of 18 men with TB in the homeless or associated populations in 2000, isolates from 15 persons had RFLP pattern 437 or 5309. These patterns differ by a one-copy addition. Thirteen of the men were long-term users of one shelter, although eight used the shelter sporadically over many years. Two patients had positive tuberculin skin tests in the past, and two other patients were clinical patients in past years (1997 and 1995). Eleven of the 15 patients were interviewed, and only one interview provided a definite epidemiologic link between two patients. Other interviews could not establish specific dates that the cases had overlapped. We reviewed 199 bed logs from the shelter for October 1999 through June 2000, a time period between documented negative tuberculin skin tests and diagnosis of active disease for three of the patients.

Of 14 homeless men in the cluster, 2 were not on the bed logs for the entire 9-month period. The remaining 12 patients were never all present on the same day. However, 11 of the 12 patients were present on 1 day in November, and 10 of the 12 were present on 6 nights in October and November 1999. Of 15 persons in the cluster (14 guests and 1 employee), we established that 13 were epidemiologically linked. Of the other two, we believe that one man’s diagnosis resulted from a false-positive culture, and the other man arrived at the shelter after June 2000 and was likely exposed later.

### Laboratory Cross-Contamination

In April and May 1994, two related instances of cross-contamination in one laboratory were identified 3 weeks apart. Five of ten cultures processed at the same time in the first instance and three of the ten in the second were submitted for RFLP analysis; they matched a strain used as a control in the decontamination and digestion procedure. The TB Division and the laboratory sent all isolates tested 2 weeks before the first instance through 2 weeks after the second instance for RFLP typing. Clinical records were reviewed, and epidemiologic follow-up was completed on all cases. No other specimens were affected.

## Discussion

We identified unexpected epidemiologic links between 21 known cases of TB (Table). However, over the course of the study, we did not unearth any new unknown infectious cases of TB by using genotyping. Two possible explanations for this lack of new cases include a historically strong programmatic interest in case finding, or conversely, the difficulty of reopening contact investigations after substantial time has elapsed.

 Although the Massachusetts TB Division has always practiced the concentric-circle method of contact investigation, RFLP typing has identified enough unexpected links and sites of transmission that we now focus on the time involved in the case-contact interaction even if that contact is not within the home or workplace. We have always emphasized the importance of considering the nonhousehold contact as a means of disease transmission, and we address the potential of the more casual contact on the basis some of the unexpected links we found (such as the student and security guard mentioned previously). Bishai et al. report that TB transmission continues despite active case finding and 15 years of directly observed therapy (20) and suggest that this transmission may have been in difficult-to-treat populations (such as the homeless) or the result of casual transmission. Other authors have also commented on the likely importance of casual transmission and the important role of RFLP typing in documentation (21–23). If the trend in Maryland noted by Bishai et al. (20) and Sterling et al. (24) is true in otherwise well-served, financially viable TB control programs, genotyping of M. tuberculosis isolates will be essential for public health interventions in these settings.

Nearly three fourths of our cases did not cluster. Although the patients with unique patterns in our dataset may have clustered with someone outside of the study (thus underestimating clustering in our population), the high numbers of unique cases still suggest that most of our cases are due to reactivation of latent TB infection. Thus, we should concentrate resources on early identification of latent TB infection and follow-up to interrupt TB transmission. In addition, the finding that our conventional contact investigations were overestimating transmission in persons born in disease-endemic countries supports the 1998 findings of Behr et al. (25). Isolates from contacts born in foreign countries were significantly more likely to have different strains than isolates from contacts born in the United States (data not shown). This finding suggests that the public health program makes certain assumptions about the definition of close contacts that are not correct in every case.

 The utility of genotyping in outbreak response cannot be overestimated. A strain designated as the genotyping network pattern 437 has been responsible for 20 cases since 1993. The strain’s reemergence in the homeless in 2000 was unsuspected, partly because men who were believed to spend few nights in homeless shelters were found to have this strain. In addition, the shelter, identified as a common site for many persons with TB, had excellent control measures. In this situation, genotyping provided evidence that warranted a thorough investigation and the development of new educational materials. Educational activities were conducted in shelters, emergency rooms, and other sites; and contacts from a large-scale screening at the site are being followed. The discovery of other unexpected clusters has resulted in involving less traditional sites in education and prevention efforts. One such educational video about TB was pilot tested in hairdressing salons in a Massachusetts community. In addition, genotyping can be applied to situations of increased cases to determine that an outbreak is not occurring, as in Hanau-Bercot et al. (8). Determining that cases have different genotypes can mean that costly mobilization of public health resources to combat an outbreak is unnecessary.

Policies to reduce cross-contamination risks have been instituted at the laboratory where cross-contamination occurred in 1994 and 1995. Live control strains are no longer used to process specimens. When a new patient’s culture grows five or fewer colonies, laboratory records are reviewed to establish the likelihood of error. However, because cross-contamination or other misclassification of a specimen or result does not rule out the possibility of a TB diagnosis, care must be taken before attributing a positive culture to error. In addition, public health programs should be able to prove or disprove laboratory contamination on a real-time basis for clinical purposes. This confirmation is important for laboratory internal quality control, can result in cost savings of several thousand dollars per misdiagnosed patient (19,26), and can save emotional and other costs to the patient.

### Limitations of Genotyping Techniques

In the cluster investigation study, 105 (66%) of 159 persons in clusters were interviewed or had epidemiologic links to everyone else in their cluster, so they were not interviewed according to the protocol. Of the remaining 54 (33%), patients refused to be interviewed (10 patients). An additional patients were defined as “administratively closed” at the end of the study. These patients did not refuse to participate in the study but could not be reached after six phone attempts or three visit attempts. Several either agreed to participate or asked to be contacted later but were unreachable. Of 45 clusters investigated as part of the official cluster investigation study with patients eligible for interview, we interviewed all persons in 13 clusters.

The RFLP technique is expensive and technically demanding; it also requires microbiologic expertise and resources to obtain cultures as well as sophisticated software for RFLP pattern comparison. Thus, turnaround time between specimen collection and availability of RFLP result may be lengthy (27). In our study, this turnaround time averaged 7 months, which may have contributed to our difficulties in obtaining patient interviews. However, identifying clusters would be a slow process even with negligible turnaround times, as latent TB infection reactivates slowly. Reopening contact investigations after receipt of RFLP results was impractical in most instances. In addition, interpreting results is more difficult because of the lack of specificity in patterns with fewer than seven IS6110 copies.

A recently reported limitation of RFLP relates to sampling (28). A certain number of cases in every population cannot be assigned RFLP types. Either the specimens do not grow M. tuberculosis, or TB is diagnosed on the basis of clinical information without bacteriological confirmation. During the study period in Massachusetts, RFLP typing provided information on 95% of our culture-confirmed cases, 77 % of our cases overall.

RFLP and any other genotyping tool can be useful for TB control if certain assumptions hold true. M. tuberculosis strains are relatively stable but not immutable; thus, if persons fall into a cluster, they probably have the same TB strain. Clustering implies recent transmission because strains change over time. A few studies (29–32) have analyzed the stability of RFLP patterns; one determined that half of the strains will demonstrate a shift in 3–4 years (29), while another study found no change (32). Neimann et al. saw almost no change in patterns among 75 isolates in chains of transmission, i.e., multiple patients (30). These studies cannot make assumptions about changes of patterns during latency, and rates of change of strains may vary. In addition, we assume that clustering in the TB control program’s population implies recent transmission and not simply endemic strains with a lack of diversity. We assume that sampling is complete and that no difference exists between the patients with or without available isolates for genotyping. For public health programs to benefit fully from a genotyping technique, it must be rapid, inexpensive, and reproducible from laboratory to laboratory. In general, the currently available DNA fingerprinting tools should be used only in conjunction with epidemiology. Universal fingerprinting, in particular, shows its greatest utility when the health department can respond to this new information by allocating additional resources to conduct investigations of unexpected clusters (33). Genotyping can then have as great an impact on public health practice nationwide as it had in Massachusetts.
